# Supplementary material for: AMPLE: An R package for capacity building on fisheries harvest strategies
Source: PLoS One. 2022 Jun 8;17(6):e0269543. doi: 10.1371/journal.pone.0269543 (PMC9176783; doi:10.1371/journal.pone.0269543)
Supplement: S1 File — The tutorial that accompanies the Introduction to HCRs app, available in the AMPLE package and through the online app. (PDF) [file pone.0269543.s001.pdf]

Introduction to Harvest Control Rules (HCRs)

Finlay Scott - OFP, SPC

2021-02-11

1 Introduction to HCRs

This tutorial is a quick introduction to Harvest Control Rules (HCRs) and their use in fisheries management. It uses the *Introduction to HCRs* Shiny app from the *AMPLE* package. There are three apps in the *AMPLE* package. This is the first one and is the one you should start with if you are unsure what an HCR is.

In this tutorial we will cover what an HCR is, how they operate, and start to think about the impact of uncertainty on HCR performance.

Note that the fishery used in this tutorial is not based on a real world fishery or stock. It's just a toy example.

2 What is an HCR?

An HCR is a pre-agreed decision rule that is used to set fishing opportunities in the future. The rule is agreed by stakeholders and managers of that fishery.

An HCR should be designed so that the management objectives of the fishery have the greatest chance of being achieved. As the real world can be a very uncertain place, a good HCR is robust to different sorts of uncertainty (which we will discuss later on).

In this tutorial we will use a simple HCR to set the catch limit of a fishery in every year. The HCR uses estimates of the current stock biomass to set that catch limit (HCRs can use information other than stock biomass but we will keep things simple for now) This rule will be applied every year in the future to set new catch limits in each year.

Note that in this app the biomass is scaled between 0 and 1, where 0 means there is no biomass (a bad thing) and 1 is the amount of biomass when is there is no fishing, i.e. the highest it can be. Scaling the biomass between 0 and 1 like this can make it easier to understand if the stock is over or underexploited.

HCRs can have different basic shapes. In this tutorial we will use a shape called a *threshold catch*. This is sometimes known as a 'hockey stick'. The catch limit is set according to the general rule shown in Figure 1.

The current stock biomass level is on x-axis along the bottom of the plot. The catch limit that will be set in the next year is on the y-axis on the side. The red line is the rule that sets the catch limit given the estimated stock biomass.

The basic idea is that if stock biomass is low the catch limit is low, and if the stock biomass is high the catch limit is high. By pre-agreeing the shape of the rule, you know exactly what the catch limit will be, given the estimated status of the stock. This is easier than all the stakeholders and managers arguing every time a catch limit needs to be set (which takes time and energy and can delay important decisions being made). Choosing the right HCR is obviously very important.

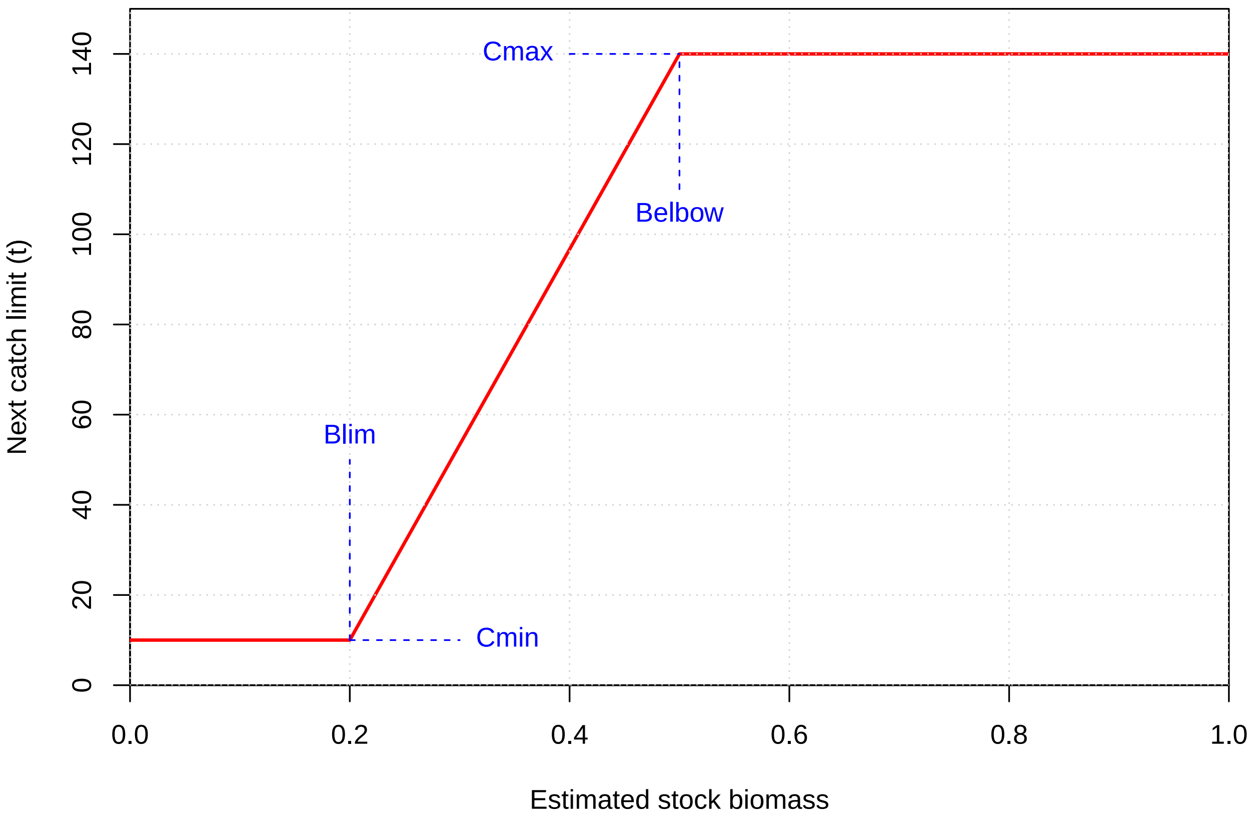

Figure 1: A threshold catch shape HCR. The shape is defined by four parameters: Cmin and Cmax (which determine the minimum and maximum catch limit) and Blim and Belbow (which determine the start and stop of sloping section).

The shape of this HCR is determined by 4 parameters: *Blim*, *Belbow*, *Cmin* and *Cmax*, shown in blue in the figure. *Cmin* and *Cmax* determine the minimum and maximum catch limit. *Blim* and *Belbow* determine the minimum and maximum biomass of the sloping section.

When the estimated stock biomass is greater than *Belbow* the catch limit is set at the maximum catch limit, *Cmax*. When stock biomass is less than *Blim* the catch limit is set at minimum catch limit, *Cmin*. When stock biomass is between *Blim* and *Belbow* the catch limit is set according to where it lies on the slope.

Important note about management procedures. An HCR is part of a management procedure (MP), along with two other elements: the data collection and the estimation process. Under a harvest strategy approach, when an MP is agreed and adopted by stakeholders all three elements of the MP are agreed together. In this tutorial, and in the app, we assume that the data collection and estimation process are the same for each HCR that we try. When we talk about comparing HCRs we are really comparing MPs.

3 Getting started

If you want to use the app online, it is available at the following address: https://ofp-sam.shinyapps.io/AMPLE-intro-hcr/

Alternatively, if you are using your own version of R and have installed the *AMPLE* package, you can run this app by entering the following commands into the R console:

```
library(AMPLE)
intro_hcr()
```

When you start the app you should see three plotting windows and a blue arrow in the main panel, and some controls in the left-hand panel (Figure 2). Your plots might look slightly different due to variations in the historic catches.

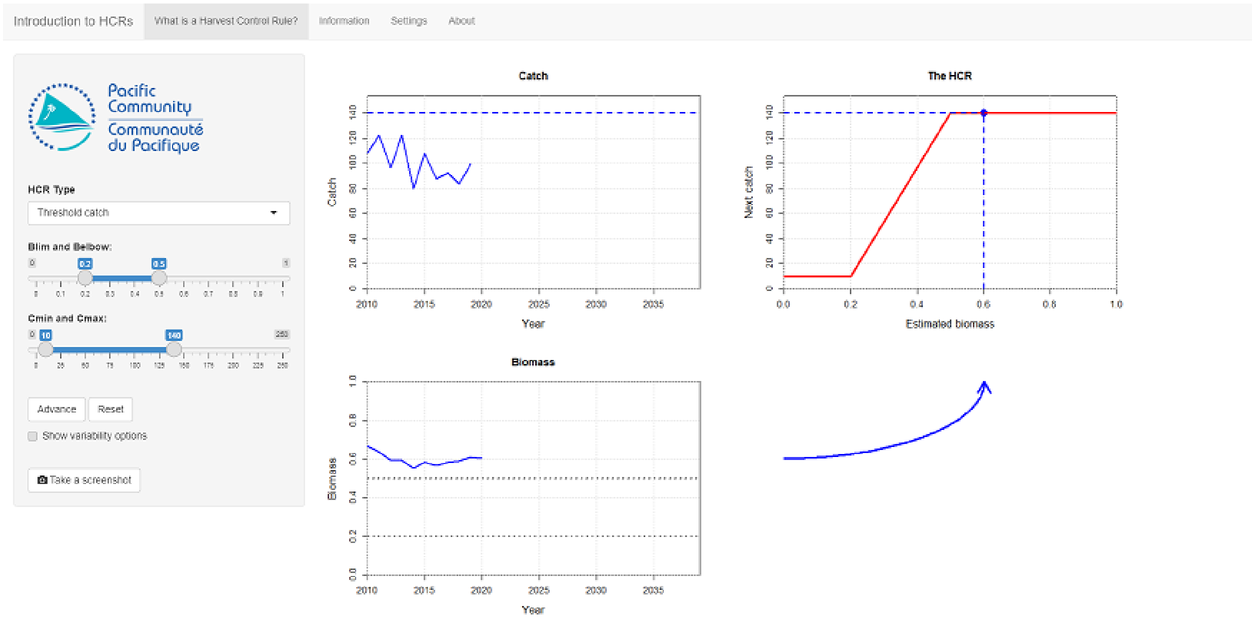

Figure 2: The opening screen of the 'Introduction to HCRs' app.

The two plots on the left-hand side of the main panel show the history of the catch and stock biomass. There are 10 years of catch history from 2010 to 2019 (the biomass has an extra year as it is the biomass at the *start* of the year). Remember that the biomass is scaled between 0 and 1, where 1 is the unfished stock biomass, i.e. the maximum biomass.

There are two horizontal dashed lines on the biomass plot. The higher one, at 0.5, is the Target Reference Point (TRP). The TRP is a management objective. Ideally, the stock biomass should be close to this line.

The lower horizontal line, at 0.2, is the Limit Reference Point (LRP). If the stock biomass falls below this level the stock is considered to be overfished and may be in serious trouble.

The HCR is shown in the top-right panel. It is the red line (ignore the blue dashed lines for the moment). As mentioned above, the shape of the HCR is determined by 4 parameters: *Blim*, *Belbow*, *Cmin* and *Cmax*. The HCR parameter values can be controlled using the sliders on the left-hand side of the screen.

The first thing to do is to set the parameters of the HCR. Set these values (if not already done): *Blim* = 0.2, *Belbow* = 0.5, *Cmin* = 10 and *Cmax* = 140. These are the default initial values, but make sure that they have been set correctly. We will refer to this HCR as *HCR 1*.

Note that as you change the HCR parameters, the shape of the HCR will change.

4 Using the HCR

The purpose of the HCR is to set the catch limit each year. The HCR uses the current estimated value of stock biomass to set the next catch limit. This rule will be applied every year in the future to set new catch limits in each year.

We start at the very beginning of 2020 and we want to use *HCR 1* to set the catch limit for 2020. The stock biomass can be seen in the bottom-left plot.

The way the HCR operates can be seen by following the blue arrow from the biomass plot to the HCR plot. The current estimated value of biomass is shown on the HCR plot as the blue dashed vertical line. This shows the same value of biomass as seen in the latest value in the biomass plot.

The catch limit in the following year is set by reading the corresponding catch limit from the HCR. This is where the blue dashed vertical line hits the red HCR line (marked by the blue dot). The new catch limit is shown by the blue dashed horizontal line on the HCR plot. The new catch limit is also shown on the catch plot at the top-left, as the blue dashed line. This represents what the catches *will* be in 2020.

We can see how the HCR works by going forward a year.

Press the **Advance** button in the left panel.

You have now advanced forward in time by a year. Several things have happened (Figure 3):

- The catch limit that was set by the HCR for 2020 has been used by the fishery. In the catch plot you should see that the catch in the year 2020 has reached this catch limit.
- The stock has responded to being fished in 2020. You should see that we now have an extra year in the biomass plot up to 2021.
- The shape of the blue arrow to the right of the biomass plot has shifted to reflect the new current estimate of biomass. The blue vertical dashed line on the HCR plot has moved left or right as well.
- The HCR has used this new value of biomass to set a new catch limit for 2021 (this may be the same value as the previous catch limit).

Press the **Advance** button again. The catch limit that was set for 2021 is used by the fishery. The stock responds to being fished again and we have a new estimate of biomass for the beginning of 2022. This new estimate of biomass is again used by the HCR to set the new catch limit for 2022 (the blue arrow will move again to reflect this).

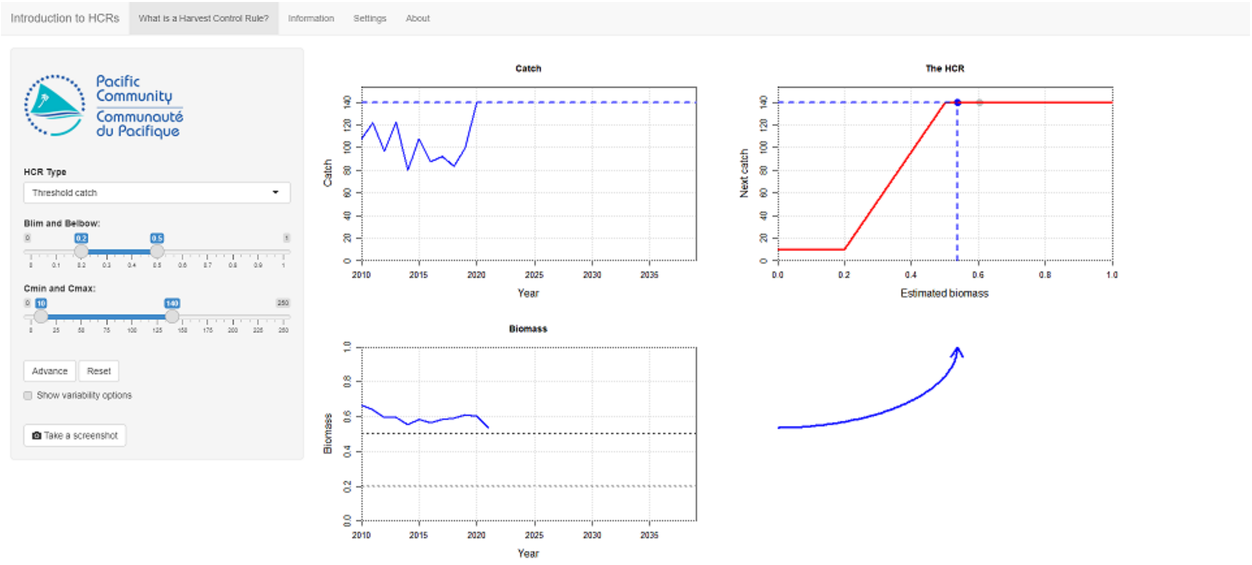

Figure 3: After pressing Advance a single time, fishing has occurred in 2020 using the catch limit that was set by the HCR. The stock biomass has been affected by the new catch limit. The HCR uses the estimate of stock biomass at the start of 2021 to set the catch limit for 2021.

Keep pressing the **Advance** button until you get to the end of the projection (in 2040). You should see the cycle:

- The HCR sets the catch limit based on the current estimate of stock biomass
- Fishing at the catch limit affects the stock biomass
- The next estimate of stock biomass is used by the HCR to set a new catch limit.

The previous values set by the HCR are shown by grey dots on the HCR plot, and the previous catch limits are shown on the catch plot as grey dashed horizontal lines.

You should see that eventually the fishery settles down to a steady catch limit and stock abundance (Figure 4).

5 Trying different HCRs

Press the **Reset** button in the left panel to clear the stock projection.

Run through the projection again by pressing the **Advance** button. Make sure that you understand how the HCR uses the latest value of biomass to set the catch limit.

Keep pressing the **Advance** button until you get to the end of the projection. In the table below write down the final value of the catch and the final value of biomass that you see on the plots. Also, note down anything interesting (if anything) that you see. For example, what is biggest change in catch that you see?

Different HCRs behave in different ways and some are better at achieving management objectives than others. The parameters of the HCR that we just tried are: $Blim = 0.2$, $Belbow = 0.5$, $Cmin = 10$ and $Cmax = 140$.

Now we try a different HCR. The general ‘hockey stick’ shape will be the same, but we will use different HCR parameters by changing the HCR parameters in the left panel.

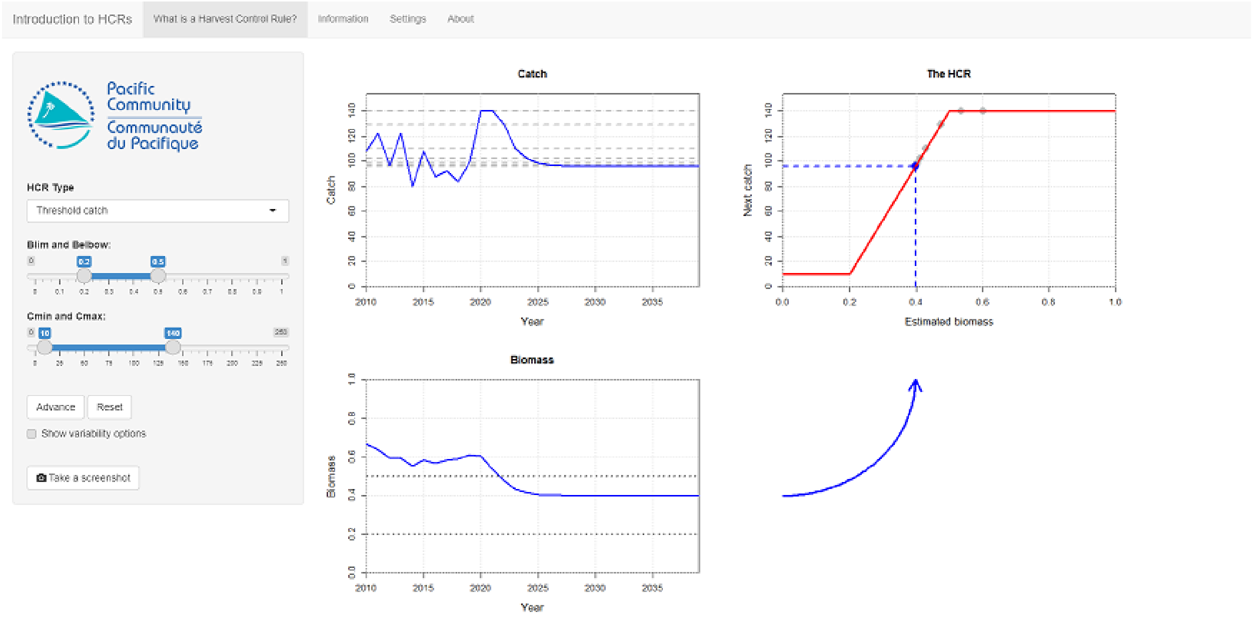

Figure 4: The results of running a full projection of HCR 1.

Change *Belbow* to be 0.3 and *Cmax* to 130. Keep the other two parameters the same. We will refer to this HCR as *HCR 2*. You should see that the HCR plot has been updated to show the new shape of the HCR.

Notice that this HCR has a lower maximum catch limit than the previous HCR. This limit will be set if the biomass is greater than 0.3 (the value of *Belbow*).

As before, repeatedly press the **Advance** button and follow the evolution of the stock and the catches (Figure 5). Note how the behaviour of the catch and biomass are different to the initial example with *HCR 1* (Figure 4).

The biology of the fish stock is *exactly* the same as the example we ran earlier with *HCR 1*. The *only* difference between the two projections is the shape of the HCR used to set future catch limits.

As before, write down the final values and any observations in the table at the end of this section.

As a final example set *Belbow* to 0.8 and *Cmax* to 150 and keep the other parameters the same. We will refer to this HCR as *HCR 3*. Notice that this HCR has a higher maximum catch limit but a much longer, shallower slope. The catch limit will only be at this maximum catch limit if the biomass is greater than 0.8 (*Belbow*).

Again, repeatedly press the **Advance** button and follow the evolution of the stock and catch limits (Figure 6). Write down the behaviour and final values in the table.

Table 1: Results from testing the three HCRs. After running a full projection, enter the final values of the biomass, the catch, and any observations you have.

HCR	Final biomass	Final catch	Anything interesting observed
HCR 1 (Belbow=0.5, Cmax=140)			
HCR 2 (Belbow=0.3, Cmax=130)			
HCR 3 (Belbow=0.8, Cmax=150)			

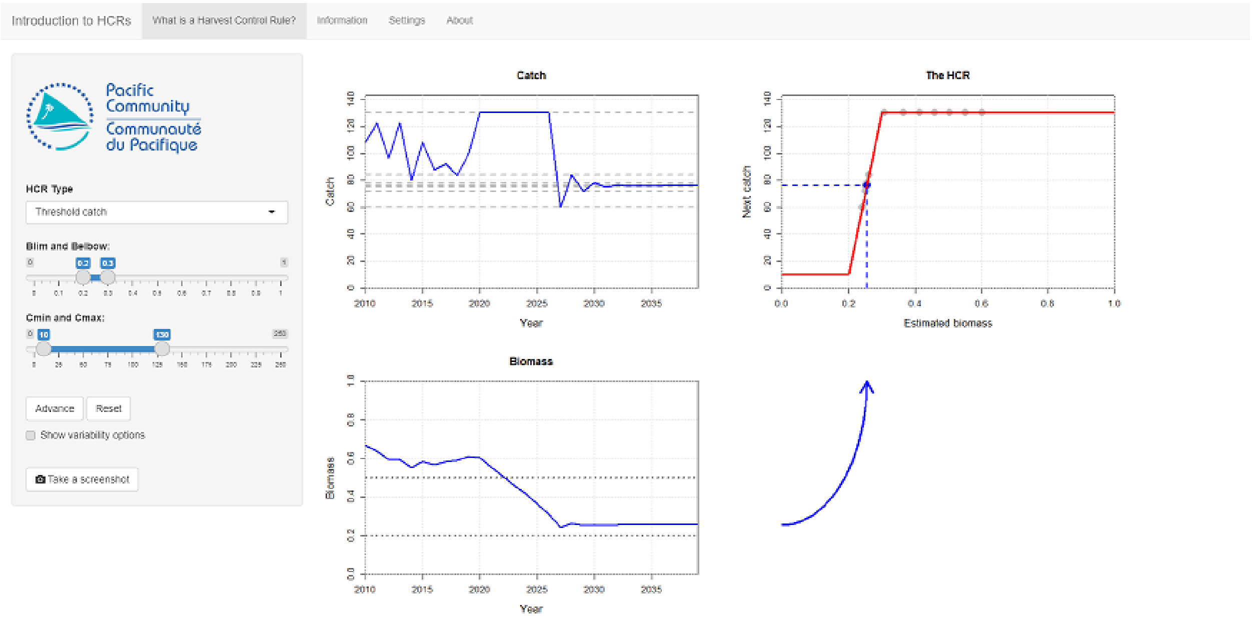

Figure 5: The results of running a full projection with HCR 2.

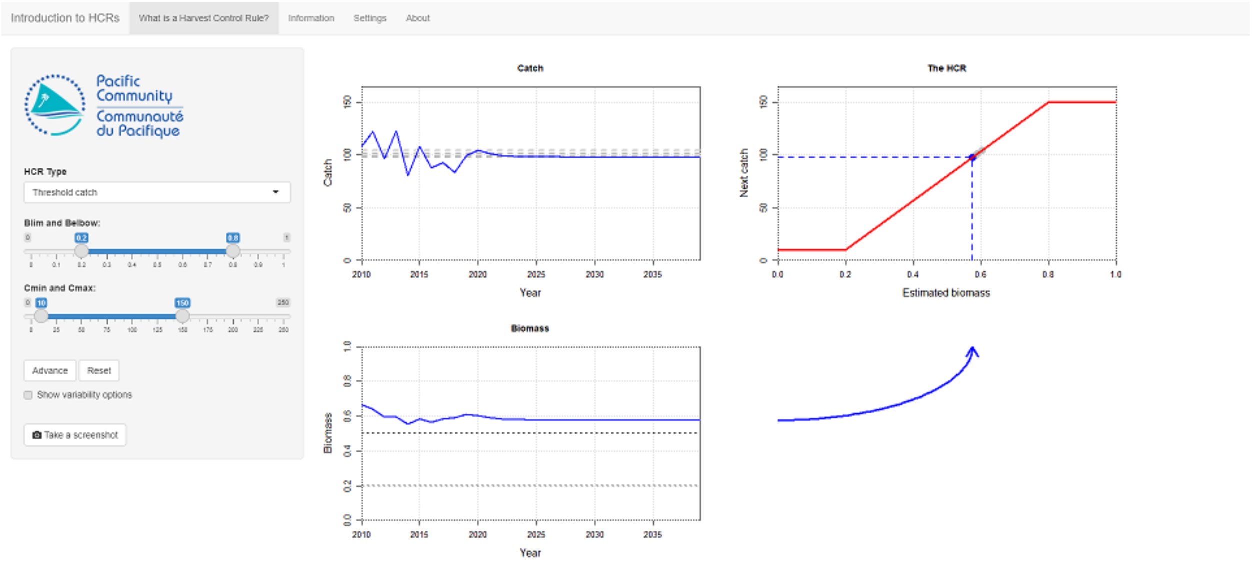

Figure 6: The results of running a full projection with HCR 3.

You should have seen that different parameterisations of the HCR lead to different fishery dynamics and final settled values.

You have now looked at the behaviour of three different HCRs.

- Which of these HCRs do you prefer and why?
- Which HCR gets the biomass closest to the TRP of 0.5?
- Which HCR results in the highest catches?
- Do any of them take the stock close to or below the LRP of 0.2?

6 Introducing uncertainty

In the real world fisheries management is affected by different types of uncertainty. By uncertainty, we mean things that we are not completely sure about, for example the exact status of the stock, and also things that are difficult to predict in the future, for example future recruitment.

The projections we have run so far have not considered uncertainty (they are known as *deterministic* simulations). This means that if we run another projection with the same HCR, the outcome will be exactly the same. Try this now and see.

It is very important to choose an HCR that is robust to different sources of uncertainty, otherwise the outcome may not be what you expected. For example, an HCR that performs well when future stock recruitment is stable may not perform well when stock recruitment varies a lot. We will look at this here.

Uncertainty is included in the app in two ways: biological variability in the stock dynamics (e.g. natural variability in recruitment) and through differences between the estimated and 'true' level of stock biomass.

These options are initially turned off. Click on the **Show variability option** option in the panel on the left to show the uncertainty options.

6.1 Biological variability

Biological variability reflects the natural variability in the stock dynamics, for example through variability in the recruitment, growth and natural mortality. It means that the future states of these biological processes are very difficult to predict with any great accuracy. Fisheries managers have no control over this source of uncertainty. As such it is very important that an adopted HCR is robust to this uncertainty.

We saw in the previous examples that, without uncertainty, eventually the projected stock biomass settles down to a constant value. What happens when we include natural variability?

Set the HCR parameters back to the values for *HCR 1* ($Blim = 0.2$, $Belbow = 0.5$, $Cmin = 10$, $Cmax = 140$). Increase the **Biological variability** option to 0.2 and project forward through time using the **Advance** button.

You should see that, unlike before, the biomass now bounces around and is not perfectly flat (Figure 7). This is because the variability in the stock biology is affecting the abundance. For example, in some years the stock may produce more recruits than in other years for the same level of biomass. This causes fluctuations in the population abundance.

As the HCR uses the estimate of biomass to set the catch limit, it means that the catch limit set by the HCR also bounces around. The catch limits set by the HCR then go onto affect the stock biomass in the same way as before.

If you press the **Reset** button and run the projection again you should see that you get a different trajectory (uncertainty in action!).

Different HCRs respond differently to biological variability. Try the following to see:

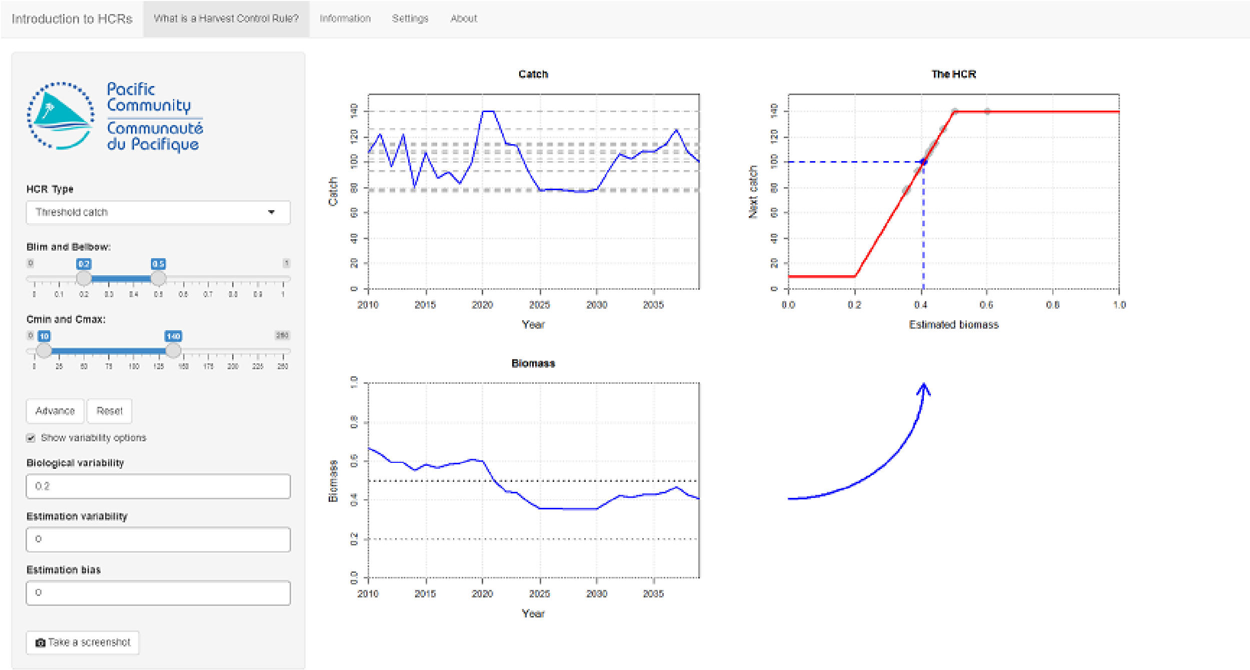

Figure 7: The results of running a full projection of HCR 1 with biological variability (note that your plot may look different).

- Set up HCR 2 again ($Belbow = 0.3$, $Cmax = 130$) and run several complete projections with the biological variability set to 0.2. What do you notice? Are the catches more or less stable than with HCR 1?
- Set up HCR 3 again ($Belbow = 0.8$, $Cmax = 150$) and run several complete projections with the biological variability set to 0.2. What do you notice?
- From this very brief experiment, which of the three HCRs do you prefer and why?

6.2 Estimation error

In the real world we do not know the true stock abundance. This means that a HCR is not driven by the *true* value of biomass. Instead it is driven by an *estimated* value (we can never know the true value). For example, the biomass can be estimated by a stock assessment model. The HCR therefore uses an *estimated* value of the stock biomass to set the next catch limit, not the *true* value. The difference between the estimated and true value of biomass is called the estimation error and it can have an important impact on the performance of a HCR.

Here estimation error is simulated in two ways: *variability* and *bias*. These can be combined.

To demonstrate these turn the **Biological variability** back to 0 and increase the **Estimation variability** to 0.2 (leave **Estimation bias** as 0 for now). This estimation variability is a crude way of simulating that we don't really know the true value of the biomass. Project the stock forward several times using the **Advance** button.

You should see that the biomass plot now shows two lines. The black one is the *true* abundance and the blue one is the *estimated* abundance. It is the estimated abundance that is used by the HCR to set the catch limit.

You should see that the stock and catch bounce around as you project forward. This variability is not caused by any biological variability (you have turned that off) but from the HCR using the uncertain *estimated* value

of biomass to set the catch limit. The catch limit, of course, affects the *real* stock abundance.

If you press **Reset** and run the same projection again by pressing **Advance** you should see a slightly different outcome. Try it.

Now turn the **Estimation variability** back to 0 and set the **Estimation bias** to 0.1. This means that the estimated value of biomass is always 10% higher than the true value, i.e. we are always overestimating the stock abundance.

Project forward and see what happens. Compare the *true* final values of biomass (the black line on the plot) and catch to the numbers you wrote down in the table above for the first HCR. Are the final values higher or lower than when there is no bias?

In the real world, both biological variability and estimation error are operating at the same time. It is very important that the selected HCR is robust to this uncertainty so that the management objectives can be achieved.

7 Summary

A HCR is a decision rule for setting future fishing opportunities. In this app, the input to the rule is the *estimated* stock biomass and the output is the catch limit in the following year.

We have looked at three different HCRs and seen that they perform differently.

Uncertainty is a big concern in fisheries management. Here we looked at biological and estimation uncertainty. We have seen that they can change the performance of the HCR and the fishery. It is very important that an HCR is robust to uncertainty. A HCR that performs well in the absence of uncertainty may not perform as well when uncertainty is present.

The big question is: how do we know which HCR to use? To answer this question, see the next tutorial *Measuring performance*.
