# Supplementary material for: AMPLE: An R package for capacity building on fisheries harvest strategies
Source: PLoS One. 2022 Jun 8;17(6):e0269543. doi: 10.1371/journal.pone.0269543 (PMC9176783; doi:10.1371/journal.pone.0269543)
Supplement: S2 File — The tutorial that accompanies the Measuring Performance app, available in the AMPLE package and through the online app. (PDF) [file pone.0269543.s002.pdf]

Measuring the performance of harvest control rules (HCRs)

Finlay Scott - OFP, SPC

2021-03-11

1 Introduction

This tutorial explores how the performance of a Harvest Control Rule (HCR) can be measured. It uses the *Measuring performance* Shiny app from the *AMPLE* package. There are three apps in the *AMPLE* package. This is the second one. If you are unsure what an HCR is you should look at the first app, *Introduction to HCRs*.

The previous tutorial (*Introduction to HCRs*) introduced HCRs and how they can be used to set future fishing opportunities based on estimates of stock status. This allowed us to explore how HCRs work and we saw that different HCRs can perform differently. We also looked at two sources of uncertainty, *biological variability* and *estimation error*, and saw that they can affect the performance of a HCR.

Before an HCR is adopted its performance is tested and evaluated using computer simulations (a process called Management Strategy Evaluation - MSE). During these evaluations, the performance of a proposed HCR is measured using a collection of indicators, known as performance indicators (PIs). The PIs should relate to the agreed management objectives of the fishery, e.g. stock sustainability, good economic performance etc. By comparing the PIs from different HCRs, the preferred HCR can be selected and put into operation. We explore comparing PIs in the third tutorial.

Fisheries management is affected by many sources of uncertainty, including uncertainty about the biology of the stock and the stock status. It is very important to understand how an HCR performs under uncertainty. Therefore, the HCR evaluations have to consider different sources of uncertainty.

In this tutorial we start to look at how PIs can be used to measure the performance of an HCR. Particular attention is paid to how uncertainty can be included in the evaluations and the effect it can have on the PI values.

To evaluate the impact of uncertainty on the performance of an HCR, many projections are performed (sometimes hundreds or even more). Each projection is known as a *replicate*. The PIs are calculated for each replicate and summaries, such as average values and ranges, are presented.

Note that the fishery used in this tutorial is not based on a real world fishery or stock. It's just a toy example.

Important note about management procedures. An HCR is part of a management procedure (MP), along with two other elements: the data collection and the estimation process. Under a harvest strategy approach, when an MP is agreed and adopted by stakeholders all three elements of the MP are agreed together. In this tutorial, and in the app, we assume that the data collection and estimation process are the same for each HCR that we try. When we talk about comparing HCRs we are really comparing MPs.

2 Getting started

If you want to use the app online, it is available at the following address: https://ofp-sam.shinyapps.io/AMPLE-measuring-performance/

Alternatively, if you are using your own version of R and have installed the *AMPLE* package, you can run this app by entering the following commands into the R console:

```
library(AMPLE)
measuring_performance()
```

When you start the app you should see something similar to the *Introduction to HCRs* app (Figure 1).

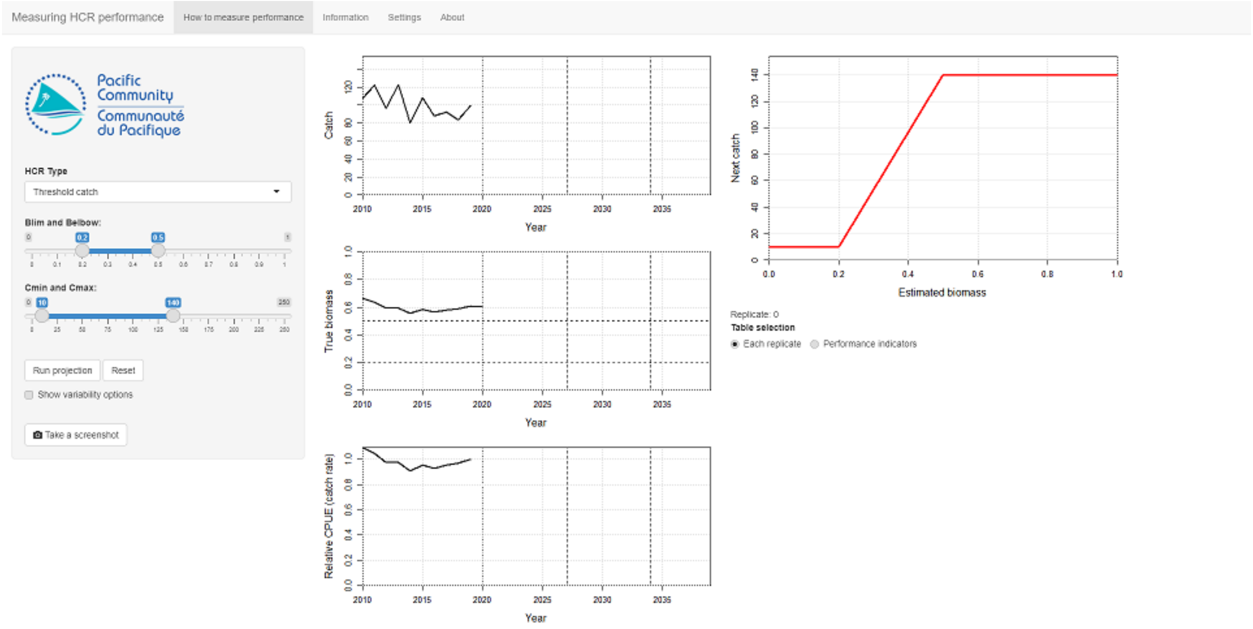

Figure 1: The opening screen of the 'Measuring performance' app.

On the left side of the main panel there are three time series plots. Two of them, catch and biomass, are similar to those seen in the *Introduction to HCRs* app (ignore the vertical dashed lines in the plots for now).

The third time series plot is a new plot of *relative CPUE*. CPUE is the catch-per-unit of effort, sometimes known as catch rate. The relative CPUE plot is the CPUE relative to the CPUE in the final historical year, 2019. The value in 2019 is therefore 1. By making the CPUE relative to some point in the past, it can be easier to interpret.

These three plots have 10 years of historical data, from 2010 to 2019. There is an additional year of data for biomass as it shows the biomass at the start of the year.

The horizontal dashed lines on the biomass plot are the Target Reference Point (TRP - 0.5) and Limit Reference Point (LRP - 0.2).

Note that in this app the biomass is scaled between 0 and 1, where 0 means there is no biomass (a bad thing) and 1 is the amount of biomass when is there is no fishing, i.e. the highest it can be. Scaling the biomass between 0 and 1 can make it easier to understand if the stock is over or underexploited.

The HCR is shown at the top of the right-hand panel. The HCR is the red line.

Underneath the HCR plot is something about **Table selection**. The option **Each replicate** should be selected. At the moment there is no table…

In the left-hand panel there are various controls, including the HCR parameters, similar to those seen in the *Introduction to HCRs* app. The initial values of the HCR parameters should be: $Blim = 0.2$, $Belbow = 0.5$,

Cmin = 10 and *Cmax* = 140. If they are not you can set them using the controls. This HCR is known as *HCR 1*.

Note that your plots might look slightly different due to variations in the historic catches.

3 Run a projection

In the previous tutorial, when you clicked the **Advance** button, you projected forward by a single year. This meant that to run a full projection you had to keep pressing the **Advance** button.

In this tutorial, when you run a projection you will project forward 20 years from 2019 to 2039 with a single button press. The HCR is applied in each year of your projection, and the corresponding catch limit used. It's exactly the same as what we were doing in the previous app, it's just that now it happens automatically without you having to press the **Advance** button lots of times.

Try this now by pressing the **Run projection** button in the left panel to perform a 20 year projection, from 2019 to 2039 (Figure 2).

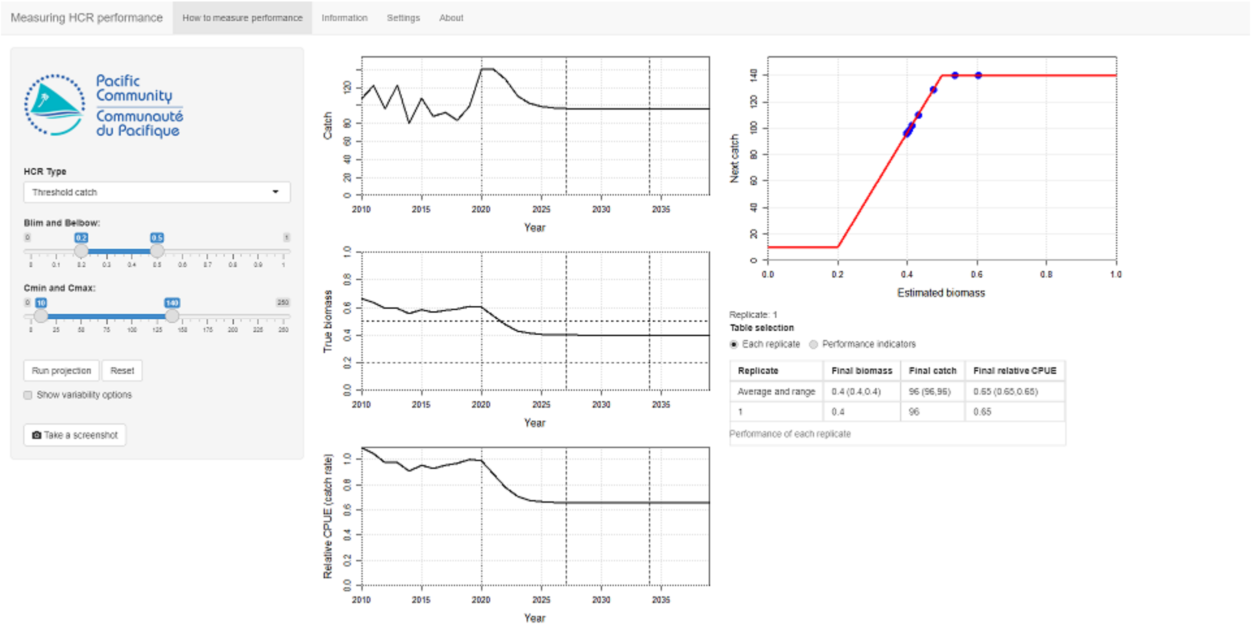

Figure 2: The result from running a single projection with no uncertainty with HCR 1.

You should see that the time series plots for catch, biomass and relative CPUE now show the full time series until 2039. The HCR was applied in each year of the projection and the HCR plot has blue points on it to show which bits were used during the projection.

With this app we will run multiple projections for the same HCR. Each projection is known as a replicate.

A table should have appeared underneath the **Table selection** control. This table shows the values of *Biomass*, *Catch* and *Relative CPUE* in the final year of the projection. We will use these as performance indicators (PIs) to evaluate the performance of HCRs.

The table has two rows. The top row gives the average value over all the replicates we have run, as well as the range of values (in brackets) in which *most* of the replicates have fallen into (strictly speaking it contains 90% of the full range of values). So far we have only run 1 replicate so this first row is not very useful right now. The results for our first replicate are shown in the second row of the table. As we run more replicates (by running more projections), more rows will appear in the table.

Click **Run projection** again. This runs *exactly the same* projection as the last one. The projection is the same as the last one because we have not included any uncertainty. Another row has appeared in the table to show the final values of the this replicate. These values are the same as the first replicate (again, because there is no uncertainty). The summary values in the first row are also exactly the same.

You may notice that the **Replicate** counter under the HCR plot also increased by one.

You can keep clicking **Run projection** to add more replicates to the table. However, as we have no uncertainty in the projection we are just running exactly the same projection over and over again with the same result.

4 Including uncertainty in the projections

Press the **Reset** button to clear out the plots and tables.

We want to know how well our HCR is going to perform under uncertainty. We introduced uncertainty in the previous tutorial (*Introduction to HCRs*). As in the previous tutorial here we have two sources of uncertainty: *Biological variability* and *Estimation error*. For the remainder of this tutorial we will only focus on biological variability (which is not to say that estimation error is unimportant - it is very important! - we're just keeping things simple at the moment).

Click on the **Show variability options** to show the uncertainty options. Set **Biological variability** to 0.2. Leave **Estimation variability** and **Estimation error bias** as 0. Note that there is nothing special about the value of 0.2, it just gives enough variability to be a useful illustration.

Keep the HCR parameters the same as before.

Click **Run projection** to project over the full time series. As we now have variability in the biological dynamics you should see that the plots are bumpy (Figure 3). This bumpiness is caused by the variability in the biological growth processes which causes variability in the stock status (the biomass). The HCR responds to the variability in the stock status by setting variable catches.

A new row will also have appeared in the replicate table that records the final values of biomass, catch and relative CPUE. These values should be different to when we ran the projections with no uncertainty.

Click **Run projection** again. You should get another line on the plots. The new line is different to the previous one (the previous one is now in grey, the new one in black). The difference between the replicates is caused by the biological variability.

Another row will also have appeared in the table to record the final values of this replicate. The final values of this second replicate will be different to the values of the first replicate. The summary row at the top of the table will have been updated.

This second projection has *exactly* the same stock and the same HCR as the first one but the outcome is slightly different. The difference is a result of the uncertainty in the biological dynamics. It is important to understand why they are different. There is a lot of natural variability in fish stocks, for example with recruitment, and it is impossible for scientists and managers to predict exactly what will happen in the future. To help understand the *possible* futures, variability is included in the biological dynamics to simulate the uncertainty.

By running many projections with uncertainty we will get slightly different results. Looking at all of the results together gives us more understanding of what might happen. This is more useful than running a single projection with no uncertainty.

Keep clicking **Run projection** until you have 10 replicates (Figure 4). More lines will appear on the plots and more rows will appear in the table. Each line will be different because each projection is different due to the biological variability.

Keep clicking **Run projection** until you get 30 or more replicates. You will see that, eventually, the average and range values in the first row of the table do not change by much with each new projection. This indicates

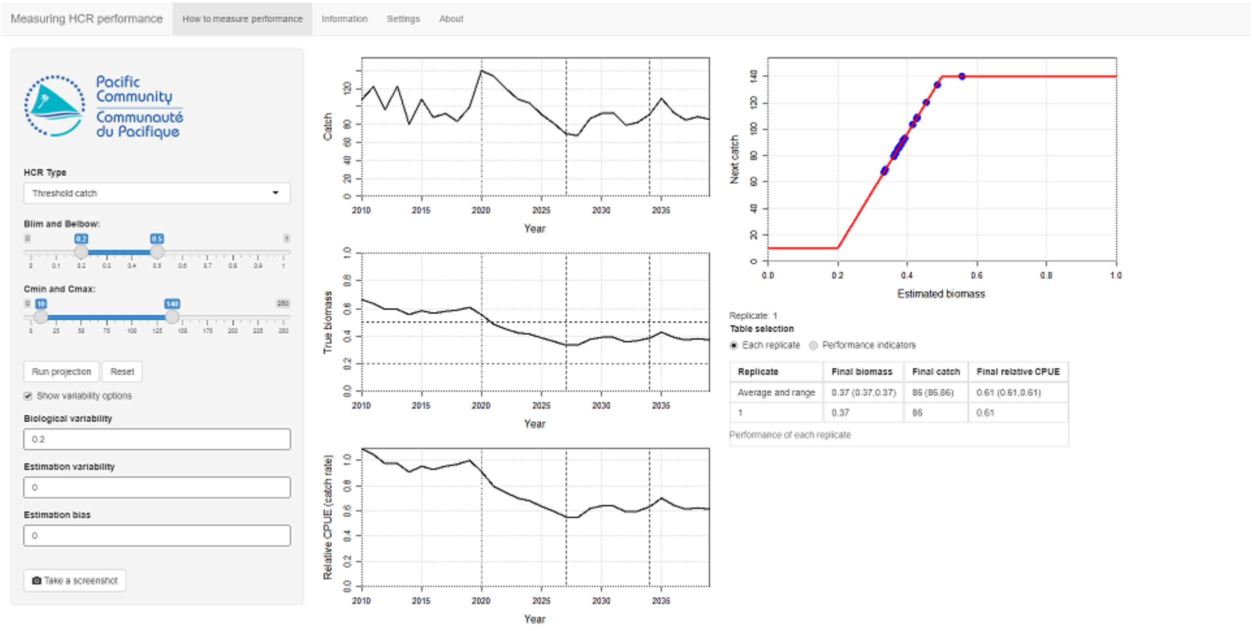

Figure 3: The result from running a single projection with biological variability with HCR 1.

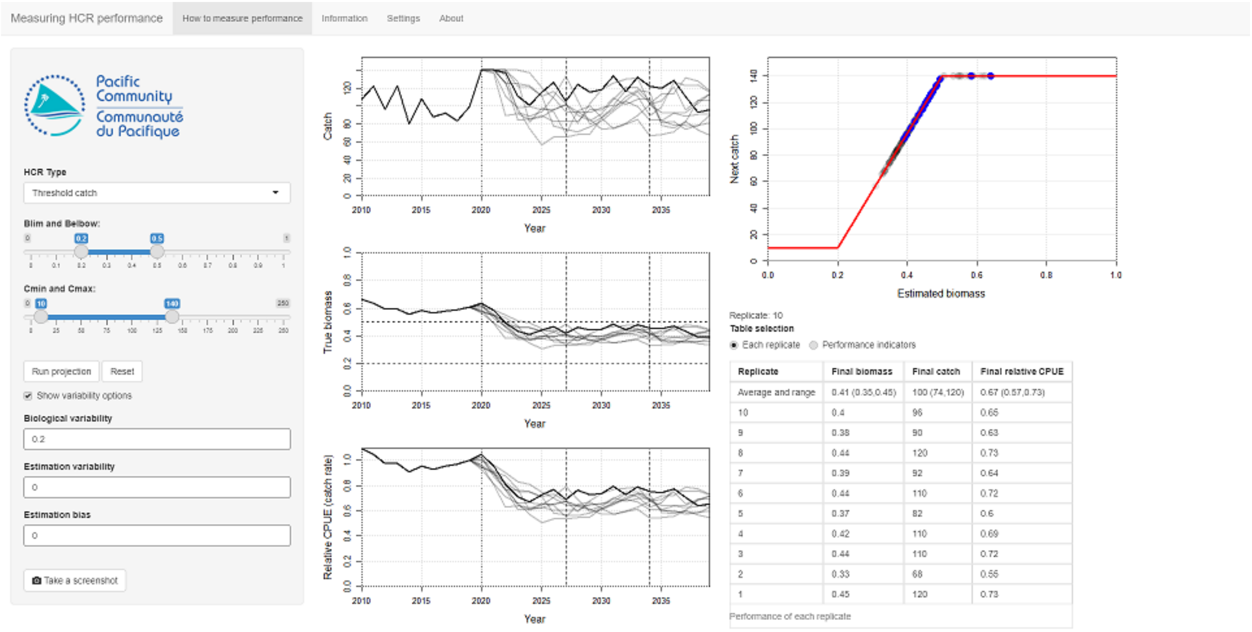

Figure 4: The result from running 10 replicates with biological variability with HCR 1.

that we are starting to understand the *distribution of expected values* from this HCR. Even though we don't know exactly what will happen in the future, we think we know the range of values that it will fall within.

This is very important when it comes to selecting a HCR for a fishery. An HCR must be robust to uncertainty, otherwise it will not perform as well as expected.

When you get to 50 or more replicates (if you click that many times...), the grey lines on the plots will be replaced by grey ribbons. The width of the grey ribbons summarises the range of most of the replicates that have been run (again, 90% of the full range - the same as the first row in the table). Using the ribbons is neater than having loads of lines on a plot. The average value and the last replicate is plotted over the ribbon for illustration purposes.

When the range of values has approximately settled down and doesn't change much as more projections are run, we can start to think about what might happen in the future.

The range of values in the plots and in the first row of the table tells us how certain we are about the future. We think that the possible *real* future will be somewhere within this range. If the range is wide, then there is a wide spread of values and we are not certain about what will happen. If the range is narrow then we have more confidence in what we think will happen.

Considering the range of values is important because sometimes it is better to choose an HCR about which we have more certainty in the future, even though the expected catches might be lower. For example, the average value of catches might be lower, but the range of possible values is small. This helps with management and planning and might be more useful than having an HCR that *might* give a high future catch but also *might* give a low future catch.

These types of projections with uncertainty are different to running a single projection with no uncertainty (known as a *deterministic* projection). If we want to understand how uncertainty may affect the performance of an HCR in the real world, running a deterministic projection is not enough.

5 Measuring performance: exercise

In this exercise you will test the performance of three different HCRs and note down the values of the three PIs: *Catch*, *Biomass* and *Relative CPUE* in the final year.

Press the **Reset** button. Use the same HCR parameters as above (*Blim* = 0.2, *Belbow* = 0.5, *Cmin* = 10 and *Cmax* = 140). This HCR is known as *HCR 1* Use the same uncertainty setttings as above (*Biological variability* = 0.2, *Estimation variability* = 0, *Estimation bias* = 0).

Run 30 replicates by pressing the **Run projection** button 30 times.

Look at the top row of the table and write down the average value and the range (the values in the brackets) of biomass, catch and relative CPUE in the final year.

For example, having just run this on my machine, for biomass I get an average value of 0.41 with a range of 0.33 and 0.45. This means that the *most likely* final value of biomass will be 0.41, but the range of possible values is from 0.33 to 0.45.

Now try a different HCR. Set these parameters: *Blim* = 0.2, *Belbow* = 0.3, *Cmin* = 10 and *Cmax* = 130 in the panel on the left. This HCR is known as *HCR 2*. Keep the same uncertainty settings.

Run 30 replicates of *HCR 2* by pressing the **Run projection** button 30 times and note down the range and average values of biomass, catch and CPUE.

Finally, do the same thing for *HCR 3*: *Blim* = 0.2, *Belbow* = 0.7, *Cmin* = 10 and *Cmax* = 150.

Table 1: Results from testing the three HCRs. After running 30 replicates, enter the average and range of values (the values in brackets) of the biomass, catch and relative CPUE in the final year.

HCR	Final biomass	Final catch	Final relative CPUE
HCR 1 (Belbow=0.5, Cmax=140)			
HCR 2 (Belbow=0.3, Cmax=130)			
HCR 3 (Belbow=0.8, Cmax=150)			

Using the results in the table, try answering these questions:

Question 1: Which of the three HCRs would you choose if you wanted to have the highest average relative CPUE in the final year?

Question 2: Which of the three HCRs would you choose if you wanted biomass in the final year to be as close as possible to the Target Reference Point (TRP) of 0.5?

Question 3: Which of the three HCRs has the highest *uncertainty* in the catch in the final year (i.e. the biggest range of catch values)?

6 Introducing performance indicators

In the exercise above we compared the values of catch, biomass and relative CPUE in the final year. We used these metrics as *performance indicators* (PIs) to evaluate the performance of the three HCRs.

Lots of different PIs are available that measure different things. For example, PIs can measure catch levels, changes in effort, the probability of biomass being above the Limit Reference Point (LRP) etc.

When comparing HCRs, the chosen PIs should relate to the management objectives for the fishery. This allows you to measure how well the fishery is performing in relation to those objectives. Some HCRs will perform well for some PIs and poorly for others. This is where the idea of prioritising PIs and evaluating trade-offs between them comes in.

In the previous exercise we only looked at what happens in the final year of the projection. We have not considered what happens during the course of the projection. When comparing HCRs we should also compare what happens in the short- and medium-term as well as the long-term.

Here we calculate PIs over three different time periods: short-, medium- and long-term. These time periods can be seen on the time series plots of catch, biomass and relative CPUE as vertical dashed lines.

Seven performance indicators are calculated (Table 2).

Table 2: The seven performance indicators used to measure performance in this app.

Performance indicator	Description
Biomass	The biomass relative to the unfished biomass.
Probability of being above the LRP	This reflects the risk of the stock being overfished.
Catch	The expected catches.
Relative CPUE	The CPUE (or catch rate) relative to the CPUE in the last historical year.
Relative effort	The fishing effort relative to the effort in the last historical year.
Catch stability	How much the catches change over time. A value of 1 means that the catches are very stable and do not change at all. A low value, close to 0, means that the catches fluctuate a lot over time (probably not a good thing).
Proximity to the TRP	How close the biomass is to the TRP on average. A value of 1 means that the biomass is always at the TRP. A lower value means that the biomass spends a lot of time being higher, or lower, than the TRP.

Generally, for most indicators, the higher the value the better (e.g. higher catches, and higher catch stability are assumed to be better than lower catches and catch levels that change a lot over time). However, higher fishing effort is not necessarily better as it may mean higher costs of fishing. Similarly, higher biomass might not be better. If the biomass is too high, it may mean you could have fished more.

7 Calculating performance indicators

We will now to start to look at the different PIs, over three time periods.

Press the **Reset** button. Set the HCR parameters to: *Blim* = 0.2, *Belbow* = 0.5, *Cmin* = 10 and *Cmax* = 140 (back to *HCR 1*). Leave the uncertainty parameters as they are (*Biological variability* = 0.2, *Estimation variability* = 0, *Estimation bias* = 0).

Select **Performance indicators** from the **Table selection** control. Now press the **Run projection** button.

As before, new lines have been added to the three time series plots. You should also see that a new table has appeared with various PIs in it. Each row in the table is a different PI. The PIs are measure over different time periods, short-, medium- and long-term. These are the table columns. The values in the table are the average of the PIs across the replicates, and the values in the brackets are the range.

It isn't very helpful to calculate the average value and the range for just 2 replicates. Keep clicking **Run projection** until you have about 50 replicates. You should see that the numbers in the table start to settle down (Figure 5).

In the previous exercise we only looked at the final values. Now, you can see the difference between the short-, medium- and long-term values. For example, you may find that the short-term catches are higher than the long-term catches. Different HCRs will perform better over different time periods and this will affect which HCR you prefer.

8 Performance indicators: exercise

We are going to look at some PIs to help us choose between the three HCRs we looked at above.

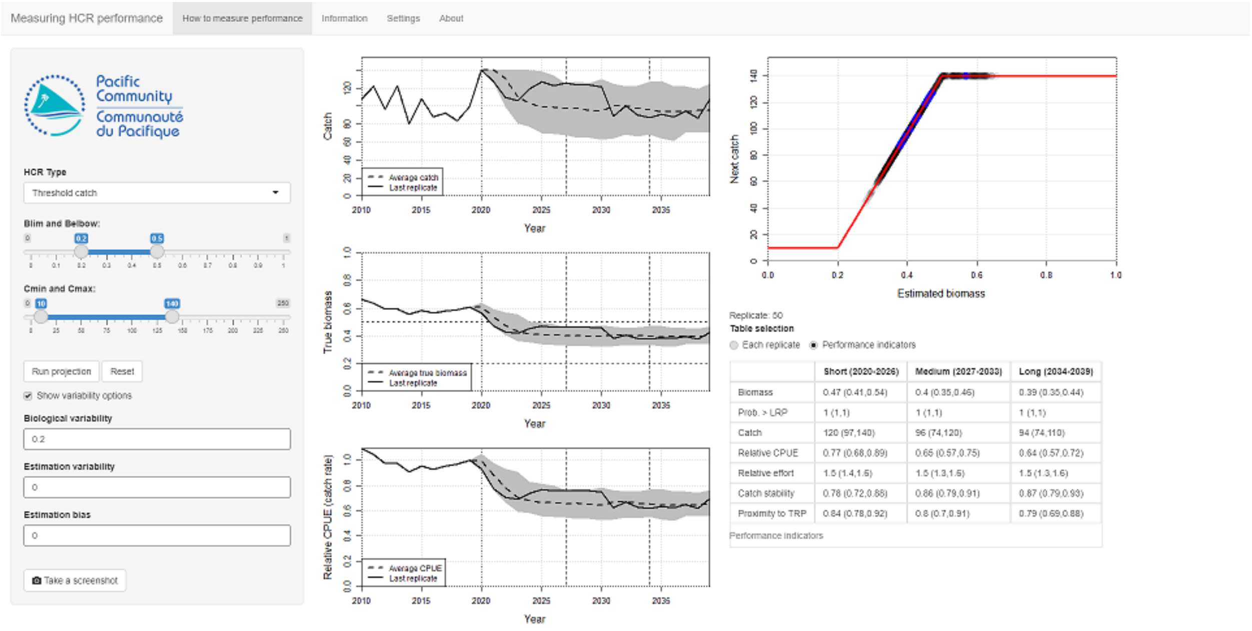

Figure 5: The performance indicators from running 50 replicates with biological uncertainty with HCR 1.

The three HCRs are:

- HCR 1 ($Blim = 0.2$, $Belbow = 0.5$, $Cmin = 10$ and $Cmax = 140$)
- HCR 2 ($Blim = 0.2$, $Belbow = 0.3$, $Cmin = 10$ and $Cmax = 130$)
- HCR 3 ($Blim = 0.2$, $Belbow = 0.7$, $Cmin = 10$ and $Cmax = 150$)

To keep things simple we are going to record the values for only three of the PIs: *Catch*, *Catch stability* and *Prob. > LRP*, in the three different time periods, in the table below (Table 3). Use the same uncertainty settings as above (*Biological variability* = 0.2, *Estimation variability* = 0, *Estimation bias* = 0).

For each HCR, run 50 iterations (by pressing **Run projection** 50, yes 50, times). I've included a line of the results that I got as an example.

Table 3: Results from testing the three HCRs. After running a 50 replicates, enter the average and range of values (the values in brackets) of the catch, catch stability and probability of being above the LRP in each time period.

HCR	Time period	Catch	Catch stability	Prob. > LRP
HCR 1	Short-term	120 (110, 130)	0.79 (0.71, 0.86)	1 (1, 1)
	Medium-term			
	Long-term			
HCR 2	Short-term			
	Medium-term			
	Long-term			
HCR 3	Short-term			
	Medium-term			
	Long-term			

When you have completed the table, try to answer these questions.

Question 1: Which HCR has the highest catches in the short-term?

Question 2: Which HCR has the highest catches in the long-term?

Question 3: Which HCR has the highest catch stability in the short-term?

Question 4: Which HCR has the highest catch stability in the long-term?

Question 5: In this example, is the indicator *Prob.* $>$ *LRP* helpful in choosing between the HCRs?

Question 6: By looking at the indicators in the different time periods, which of the HCRs do you prefer and why?

9 Summary

In this tutorial we started to use performance indicators (PIs) to measure the performance of different HCRs. The indicators allow us to compare the performance of candidate HCRs. The performance in different time periods is important (it's not just the final destination, but the journey).

Additionally, HCRs need to be robust to uncertainty. In this tutorial we have seen that when we include uncertainty in an HCR evaluation, it is necessary to run many projections to allow us to understood how the HCR is going to perform.

In this tutorial we have 7 PIs over 3 time periods giving us a total of 21 PIs to consider. Each PI is reported as an average value plus a range. This is a lot of information to process! You can see that considering more and more indicators can quickly lead to an overwhelming amount of information. How we try to understand all of it is covered in the next tutorial.
