# Supplementary material for: AMPLE: An R package for capacity building on fisheries harvest strategies
Source: PLoS One. 2022 Jun 8;17(6):e0269543. doi: 10.1371/journal.pone.0269543 (PMC9176783; doi:10.1371/journal.pone.0269543)
Supplement: S3 File — The tutorial that accompanies the Comparing Performance app, available in the AMPLE package and through the online app. (PDF) [file pone.0269543.s003.pdf]

Comparing the performance of Harvest Control Rules (HCRs)

Finlay Scott - OFP, SPC

2021-04-11

1 Introduction

This tutorial explores how to compare the performance of several Harvest Control Rules (HCRs). It uses the *Comparing performance* Shiny app from the *AMPLE* package. There are three apps in the *AMPLE* package. This is the third one. If you are unsure what an HCR is you should look at the first app, *Introduction to HCRs*.

The previous tutorial (*Measuring the performance of HCRs*) introduced the idea of performance indicators (PIs) and how they can be used to measure the performance of an HCR in relation to the management objectives. The performance of a proposed HCR is tested and evaluated using computer simulations (known as Management Strategy Evaluation - MSE). During these evaluations the performance is measured using a collection of PIs.

In this tutorial we will generate PIs for several candidate HCRs and compare them. By considering what the objectives of the fishery are, we can then select the most appropriate HCR.

Note that the fishery used in this tutorial is not based on a real world fishery or stock. It's just a toy example.

Also **note** that in this app the biomass is scaled between 0 and 1, where 0 means there is no biomass (a bad thing) and 1 is the amount of biomass when is there is no fishing, i.e. the highest it can be. Scaling the biomass between 0 and 1 can make it easier to understand if the stock is over or underexploited.

Important note about management procedures. An HCR is part of a management procedure (MP), along with two other elements: the data collection and the estimation process. Under the harvest strategy approach, when an MP is agreed and adopted by stakeholders all three elements of the MP are agreed together. In this tutorial, and in the app, we assume that the data collection and estimation process are the same for each HCR that we try. When we talk about comparing HCRs we are really comparing MPs.

2 Getting started

If you want to use the app online, it is available at the following address: https://ofp-sam.shinyapps.io/ample-comparing-performance/

Alternatively, if you are using your own version of R and have installed the *AMPLE* package, you can run this app by entering the following commands into the R console:

```
library(AMPLE)
comparing_performance()
```

When you start the app you should see something similar to the *Measuring performance* app (Figure 1).

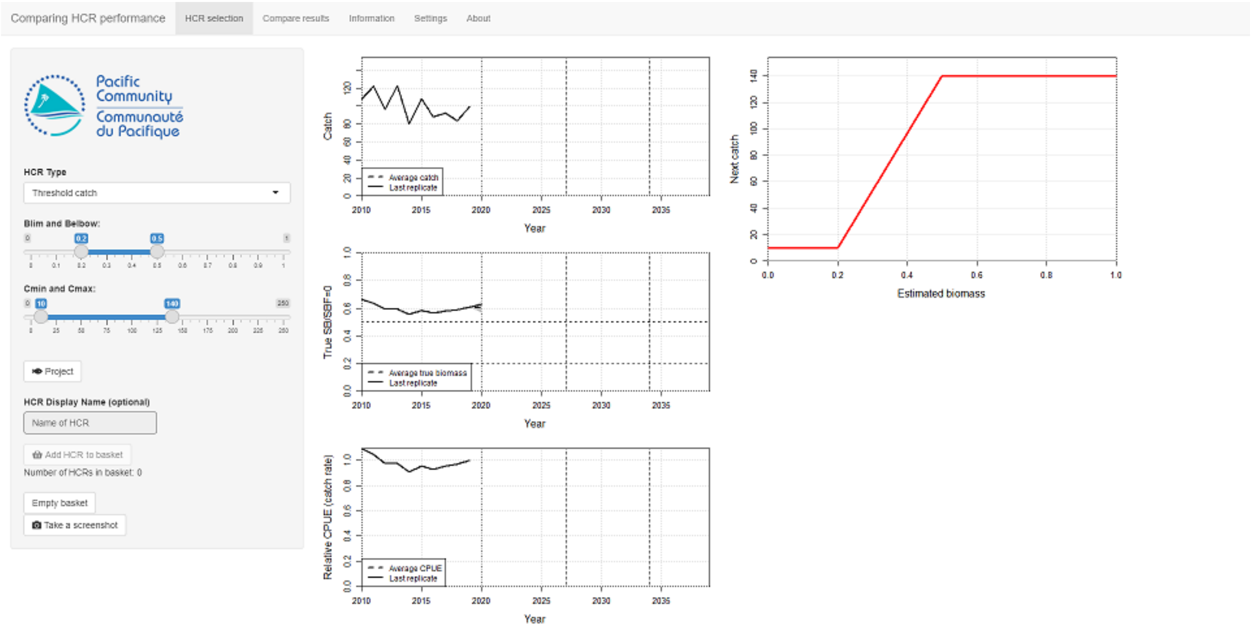

Figure 1: The opening screen of the 'Comparing performance' app.

On the left side of the main panel there are plots of catch, biomass and CPUE relative to the CPUE in the last historical year (2019) These three plots have 10 years of historical data, from 2010 to 2019. There is an additional year of data for biomass as it shows the biomass at the start of the year.

The horizontal dashed lines on the biomass plot are the Target Reference Point (TRP = 0.5) and Limit Reference Point (LRP = 0.2).

The HCR is shown at the top of the right-hand panel. The HCR is the red line.

Underneath the HCR plot is currently blank…

In the left-hand panel there are various controls, including the HCR parameters. The initial values of the HCR parameters should be: $Blim = 0.2$, $Belbow = 0.5$, $Cmin = 10$ and $Cmax = 140$. If they are not you can set them using the controls in the left panel. This HCR is known as *HCR 1*.

Note that your plots might look slightly different due to variations in the historic catches.

In this app, *biological variability* is already switched on and set to a value of 0.2. You can change the variability options by looking in the **Settings** tab (but don't do this yet).

3 Evaluating the performance

We are going to compare the performance of several HCRs. We will do this by running projections with uncertainty for different HCRs and calculating a range of performance indicators (PIs) for them. The PIs of the different HCRs can then be compared in different ways.

The basic process we will follow is:

- Set up an HCR using the HCR parameters on the left-hand side.
- Project the stock forward in time under that HCR (by pressing the **Project** button).
- Have a quick check of the resulting time series plots and PI values.

- If you like the HCR, add it to the basket of candidate HCRs (by pressing the **Add HCR to basket** button).
- When you have several HCRs in the basket, go to the **Compare performance** tab at the top of the screen and take a look at their relative performance.

3.1 Evaluating the first HCR

The initial values of the HCR parameters should be: *Blim* = 0.2, *Belbow* = 0.5, *Cmin* = 10 and *Cmax* = 140. If not, set these parameters. This HCR will be referred to as *HCR 1*.

Press the **Project** button to run the projection. In the previous tutorial when you ran the projection it ran a single projection. Here, when you run a projection 250 replicates will be run (i.e. 250 projections will be run). This may take a few seconds, depending on your machine. As noted above, biological variability is switched on and set to 0.2 so that each replicate is slightly different. This is the same as using the *Measuring performance* app and clicking **Project** 250 times.

After clicking **Project** the time series plots will show the results. The grey ribbon shows the range of values of the replicates (it contains 90% of the full range of values). The average value (the dashed line), and the final replicate as an example (the solid line) are also shown.

A table of performance indicators has also appeared underneath the HCR plot. Here we calculate PIs over three different time periods: short-, medium- and long-term. These time periods can be seen on the time series plots of catch, biomass and relative CPUE as vertical dashed lines.

Seven performance indicators are calculated (Table 1).

Table 1: The seven performance indicators used to measure performance in this app.

Performance indicator	Description
Biomass	The biomass relative to the unfished biomass.
Probability of being above the LRP	This reflects the risk of the stock being overfished.
Catch	The expected catches.
Relative CPUE	The CPUE (or catch rate) relative to the CPUE in the last historical year.
Relative effort	The fishing effort relative to the effort in the last historical year.
Catch stability	How much the catches change over time. A value of 1 means that the catches are very stable and do not change at all. A low value, close to 0, means that the catches fluctuate a lot over time (probably not a good thing).
Proximity to the TRP	How close the biomass is to the TRP on average. A value of 1 means that the biomass is always at the TRP. A lower value means that the biomass spends a lot of time being higher, or lower, than the TRP.

Generally, for most indicators, the higher the value the better (e.g. higher catches, and higher catch stability are assumed to be better than lower catches and catch levels that change a lot over time). However, higher fishing effort is not necessarily better as it may mean higher costs of fishing. Similarly, higher biomass might not be better. If the biomass is too high, it may mean you could have fished more.

By looking at the summary plots and the table of PIs, we can decide if we think the current HCR is worth considering further.

The HCR we have just tested, *HCR 1*, looks OK (e.g. there is a high chance of being above the LRP and the expected catches look OK). We therefore consider *HCR 1* as a *candidate* HCR, i.e. a candidate for adoption.

Click on the **Add HCR to basket** button to add the HCR to the basket of candidate HCRs. You should see that the counter **Number of HCRs in basket** increases by 1.

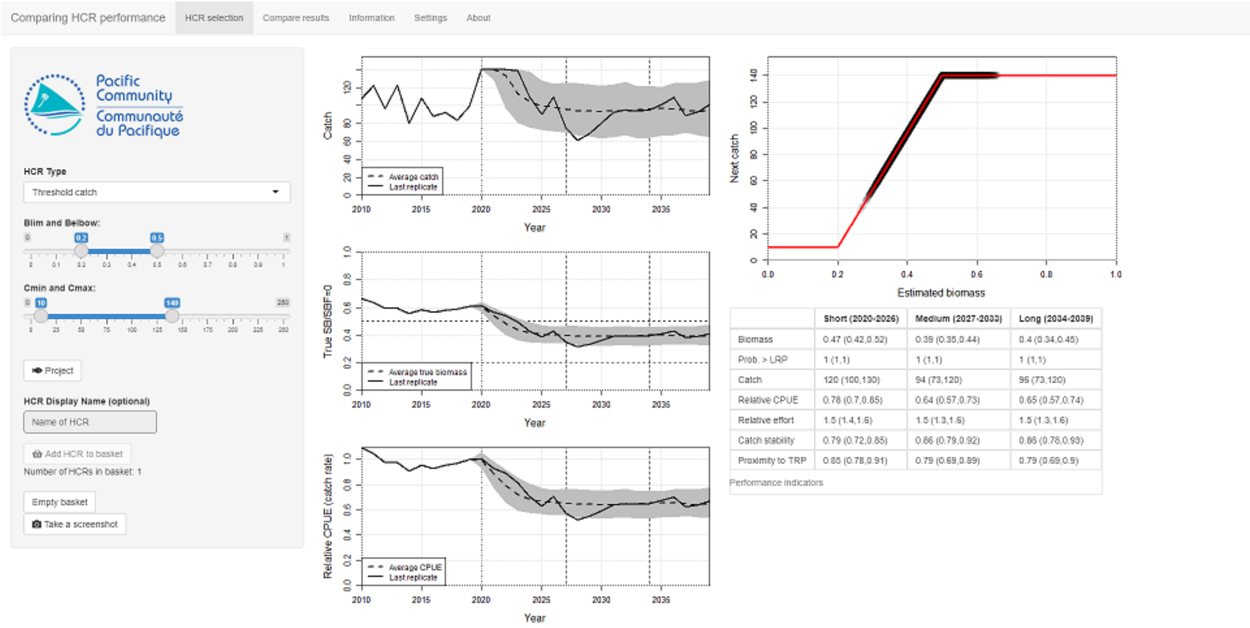

Figure 2: The results from evaluating HCR 1.

3.2 Add more HCRs to the basket

We now have one HCR in our basket. We are going to add two more HCRs to the basket and then compare the results of all three of them.

Repeat the above process (set up the HCR, run the projection, add to the basket) for two more HCRs. Use the following parameters:

- HCR2: $Blim = 0.2$, $Belbow = 0.3$, $Cmin = 10$ and $Cmax = 130$
- HCR3: $Blim = 0.2$, $Belbow = 0.7$, $Cmin = 10$ and $Cmax = 150$

You should now have three HCRs in your basket (check the counter and don't forget to **Add HCR to basket** after projecting).

4 Comparing the performance

We can now compare the performance of the three HCRs.

Select the **Compare performance** tab at the top of the app window. In the main panel you should see a lot of bar plots.

Each window shows a different PI for each of the HCRs in the basket. Each HCR has a different colour. The height of the bars is the average value of that PI. The bars are grouped into the three different time periods (short-, medium- and long-term) (Figure 3).

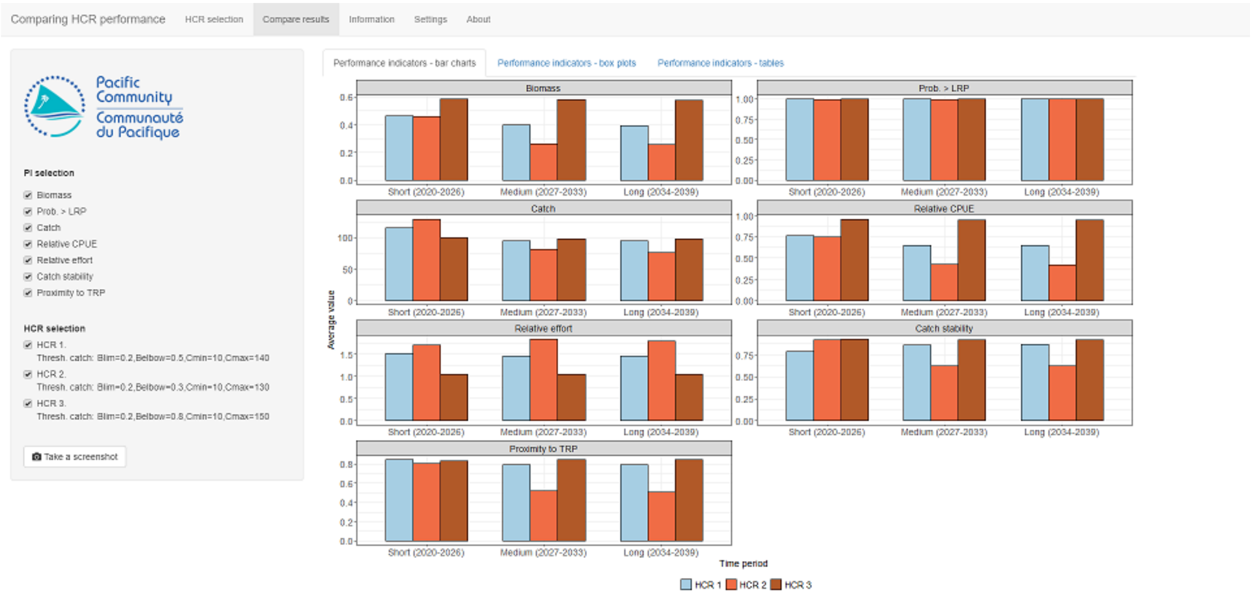

Figure 3: Using bar charts to compare the average values of performance indicators of three candidate HCRs.

We are looking at the average values of seven PIs for three HCRs in three different time periods. This is a lot of information! We want to be able to choose which HCR best fits our objectives but it can be difficult when there is so much to look at.

To make things easier, we can drop PIs that we think are unimportant (perhaps they do not measure anything related to your management objectives) by deselecting them from the list in the left panel. Bear in mind, that a PI that may be unimportant to you, may actually be very important to someone else.

Similarly, candidate HCRs can be deselected if they do not meet the objectives.

For example, we might think that the *Biomass*, *Catch stability* and *Relative effort* are less important than the other PIs. Deselect them from the list on the left. This still gives us four PIs left to consider (Figure 4).

The results for the probability of being above LRP look pretty similar for the three HCRs. They all show a high probability of being above the LRP. This means that this indicator is not providing us with any information to help us choose between the HCRs (note that it may be helpful for other HCRs). We can deselect it, leaving us with just three PIs to consider.

Looking at the performance of *HCR 2*, we can see that in the short-term the average catches are higher than *HCR 1* and *HCR 3*. However, in the medium- and long-term it has the lowest catches. Additionally, it has the lowest relative CPUE and lowest 'proximity to the TRP' (i.e. it is the furthest away from the TRP) in all three time periods. Unless high catches in the short-term is the absolute top priority, we can decide that *HCR 2* does not meet our management objectives as well as *HCR 1* and *HCR 3*. Deselect *HCR 2* from the list on the left (Figure 5).

We now have only two HCRs to consider. The choice between *HCR 1* and *HCR 3* depends on what objectives you have. This will depend on your *priorities* and what *trade-offs* you are prepared to accept.

Considering catches, *HCR 1* has slightly higher average catches in the short-term. The average catches in the medium- and long-term are similar for both HCRs. *HCR 3* has higher average relative CPUE in all three time periods. *HCR 3* also gets you slightly closer to the TRP in the medium- and long-term.

These are the kind of decisions that managers and stakeholders will need to make. You should always have your management objectives in the back of your mind when reviewing the results.

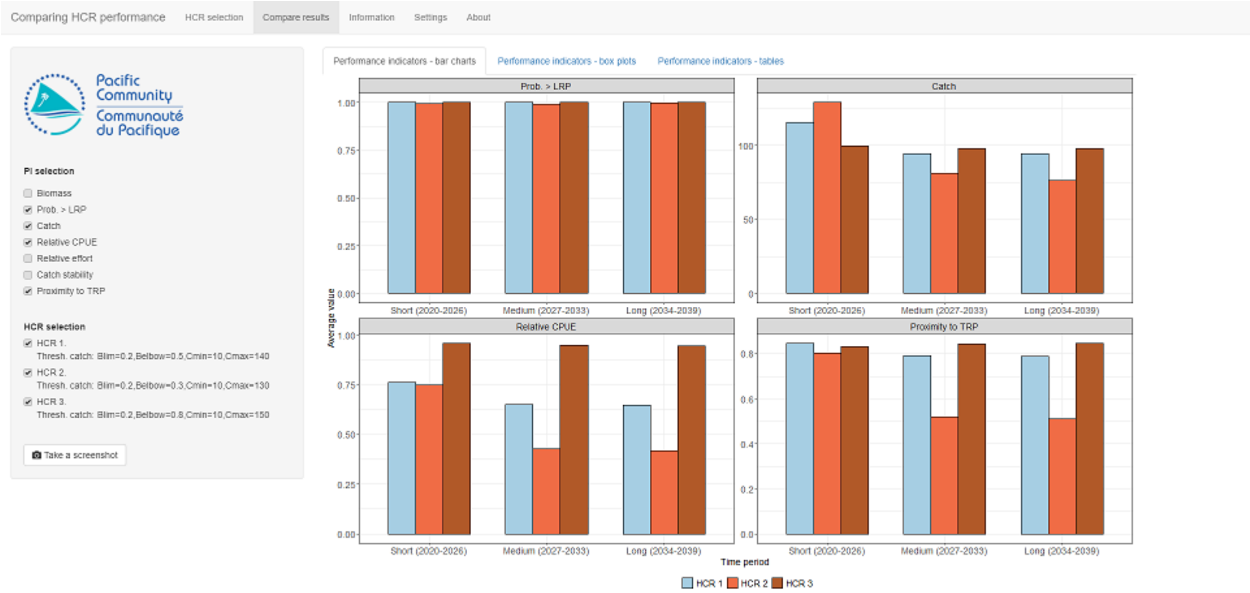

Figure 4: Using bar charts to compare the average values of only four performance indicators of three candidate HCRs.

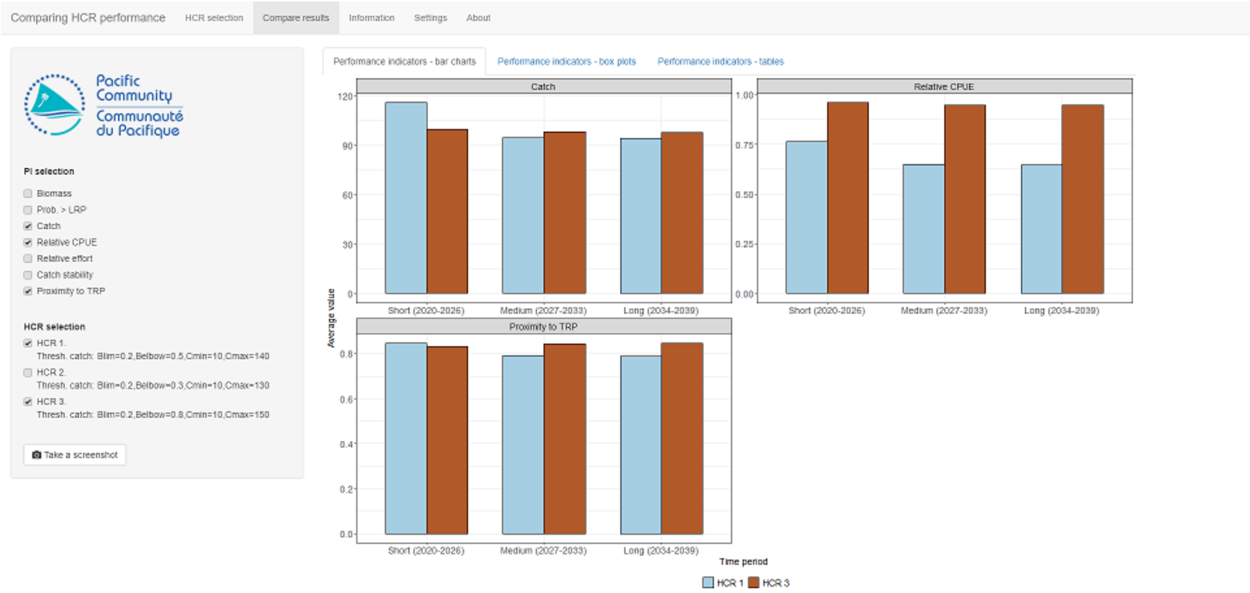

Figure 5: Using bar charts to compare the average values of only three performance indicators after dropping HCR 2.

4.1 Box plots

So far, we have only used the bar charts that show the *average* values of the PIs. This is only part of the story.

Click on the tab at the top that says **Performance indicators - box plots**. Box plots give you the *range* of expected values of each indicator, and hence the **uncertainty** in their value. The taller the box, and the longer the whiskers, the greater the range of expected values. The black line across the middle of each box is the average value, the same that is in the bar charts in the previous tab (Figure 6).

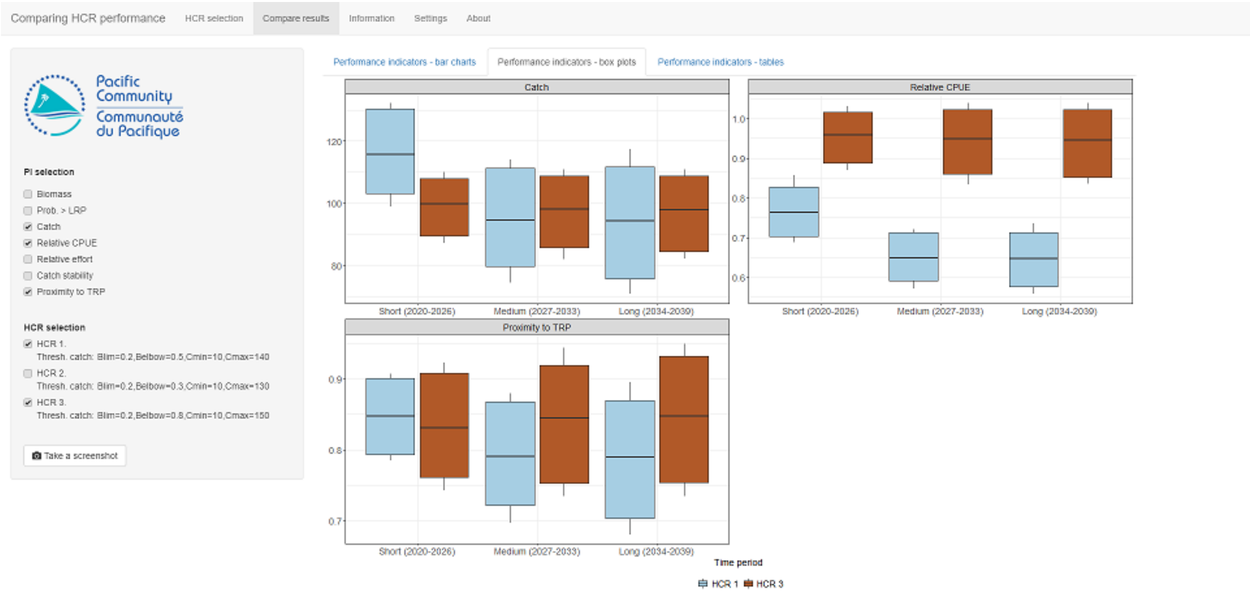

Figure 6: Using box plots to compare the range of expected values of three performance indicators for HCR 1 and HCR 3.

For example, the average value of the catches in the medium- and long-term is about the same for both HCRs (the black line in the middle is at the same height). However, the box for *HCR 1* is bigger than that of *HCR 3*. This means that, although the average value might be the same, we are *less certain* about what the catches will actually be under *HCR 1*. This can be an important factor when considering which HCR you prefer.

4.2 Table

Finally, it is possible to see all the indicators for each HCR in a table (click on the **Performance indicators - tables**. This might not look as pretty, but can be very useful for really digging down into the results, particularly if you have narrowed your choices to just a few HCRs and PIs.

5 Exercises

Return to the *HCR selection* tab at the top, click the *Empty basket* button (and confirm that you are happy to do that). This will empty all the HCRs from your basket.

5.1 Exercise 1

Using a similar process as above, find an HCR that meets the following two conditions:

- Gives the highest possible average catches in the short-term and
- Always has a probability of the biomass being above the LRP of at least 0.8.

Try out as many HCRs as you want (5, 10, 20. . . , keep adding them to the basket), then use the different methods for exploring the performance to select the best one.

5.2 Exercise 2

Now find an HCR that meets these two conditions:

- Gives the highest possible average relative CPUE in the long-term and
- Maintains average catches above 100 in all time-periods.

Write down your final HCR parameter settings and also why you think it is the best compared to the others.

6 Summary

Choosing a preferred HCR is not a simple task. It is possible to calculate many different performance indicators to measure their performance. The ranges of the indicator values should be considered as well as their average value. Additionally, you can have different time periods to consider.

It may not be possible to find a HCR that performs well for all the chosen PIs. In this case PIs should be considered in order of their priority and trade-offs will need to be evaluated.
